# Characterization of the complete chloroplast genome of *Clematoclethra scandens* subsp. *actinidioides* (Actinidiaceae)

**DOI:** 10.1080/23802359.2022.2110532

**Published:** 2022-08-29

**Authors:** Lei Zhang, Ying Zhang, Yun Jia, Fangbing Ding, Fengwei Wang, Gang Yu, Yongpeng Wu

**Affiliations:** a Shaanxi Engineering Research Centre for Conservation and Utilization of Botanical Resources, Xi’an, China; b Xi’an Botanical Garden of Shaanxi Province (Institute of Botany of Shaanxi Province), Xi’an, China

**Keywords:** Chloroplast genome, *Clematoclethra scandens* subsp. *actinidioides*, phylogenetics

## Abstract

*Clematoclethra scandens* subsp. *actinidioides* (Actinidiaceae) is an endemic medicinal species in China. Here, we first sequenced and characterized the complete chloroplast genome of *C. scandens* subsp. *actinidioides*. The chloroplast genome was 159,341 bp in length, containing a large single-copy of 88,351 bp and a small single-copy of 21,580 bp separated by a pair of identical inverted repeat regions of 24,705 bp each. A total of 131 genes were identified, including 84 protein-coding genes, 39 tRNA, and eight rRNA genes. The phylogenetic analysis of *C. scandens* subsp. *actinidioides* showed a relatively close relationship with *Clematoclethra scandens* subsp. *hemsleyi*.

*Clematoclethra scandens* subsp. *actinidioides* (Maximowicz) Y. C. Tang & Q. Y. Xiang 1890 is an endemic medicinal plant in the family Actinidiaceae, which is distributed in the temperate and subtropical regions in central and western China (Li et al. 2007; Yang et al. 2014). The roots of *C. scandens* subsp. *actinidioides* have long been used as an important traditional medicinal to treat chronic hepatitis, rheumatic arthritis, and hernia (Song et al. 2001). Previous studies have focused on the chemical composition, pollen morphology, and taxonomy for this species (Yang et al. 2014; Xiao et al. 2015). Due to its various flavonoids and triterpenoids, *C. scandens* subsp. *actinidioides* not only has a high medicinal value, but also has scientific research value as an endemic species (Xiao et al. 2015). Herein, we first sequenced and assembled the complete chloroplast genome of *C. scandens* subsp. *actinidioides* and analyzed its phylogenetic relationship.

The fresh leaves from a wild single tree of *C. scandens* subsp. *actinidioides* were collected from Feng River, Shaanxi Province (108°48′16.76″E, 33°50′22.77″N) and the voucher specimens were stored at Xi’an Botanical Herbarium under accession number XBH20200822 (http://www.xazwy.com/; Yongpeng Wu, Email: 43566351@qq.com). Total genomic DNA was extracted using CTAB method (Doyle and Doyle 1987) and sequenced with Illumina Hiseq 4000 platform. The chloroplast genome was de novo assembled using Novoplasty (Dierckxsens et al. 2019). The annotation was performed with the online annotation tool CPGAVAS2 (Shi et al. 2019). Phylogenetic analyses were carried out by maximum likelihood (ML) using MEGA v7.0 (Kumar et al. 2016) with 1000 bootstrap replicates.

The chloroplast genome of *C. scandens* subsp. *actinidioides* was a typical quadripartite circular molecule with a length of 159,341 bp, including a large single-copy region (LSC) of 88,351 bp and a small single-copy region (SSC) of 21,580 bp, and two 24,705 bp inverted repeat regions (IRs). A total of 131 genes were annotated, containing 84 protein-coding genes, 39 tRNA genes, and eight rRNA genes. Unexpectedly, we observed the chloroplast genome lacks *clpP* gene, which is consistent with *C. scandens* subsp. *hemsleyi* chloroplast genome in the genus *Clematoclethra* (Wang et al. 2016). The overall GC content of *C. scandens* subsp. *actinidioides* plastid genome is 38.3%, while the corresponding values of LSC, SSC, and IR regions are 38.9%, 37.1%, and 37.5%, respectively.

To confirm the phylogenetic position of *C. scandens* subsp. *actinidioides*, 14 chloroplast genome sequences of Actinidiaceae, Lardizabalaceae, and Passifloraceae were aligned by MEGA v7.0 (Kumar et al. 2016). The result indicated that *C. scandens* subsp. *actinidioides* was found to be relatively closely related to *C. scandens* subsp. *hemsleyi* chloroplast compared to other species of *Actinidia* genera in Actinidiaceae (Figure 1). The chloroplast genome information reported in this study provided fundamental data for the bioinformatics and systematics of the Actinidiaceae.

**Figure 1.**
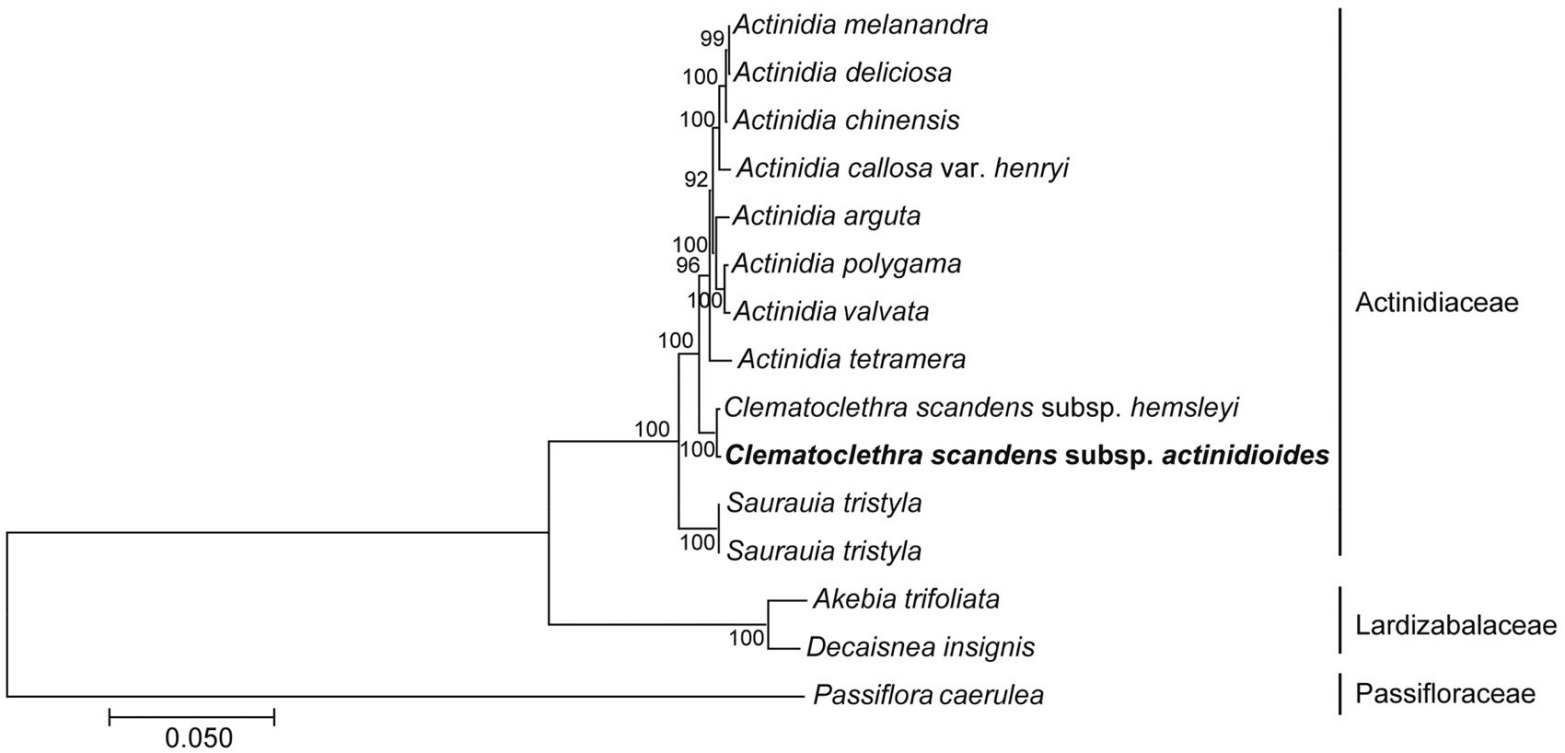
Phylogenetic tree produced by maximum-likelihood (ML) analysis based on 15 chloroplast genome sequences. The following sequences were used: *Actinidia melanandra* MK863365 (Zhao et al. 2019), *Actinidia deliciosa* NC026691, *Actinidia chinensis* NC026690, *Actinidia callosa* var. *henryi* NC043861 (Wu et al. 2019), *Actinidia arguta* NC034913, *Actinidia polygama* NC031186 (Wang et al. 2016), *Actinidia valvata* NC050357 (Wang et al. 2016), *Actinidia tetramera* NC031187 (Wang et al. 2016), *Clematoclethra scandens* subsp. *hemsleyi* KX345299 (Wang et al. 2016), *Saurauia tristyla* MG912839, *Saurauia tristyla* NC044098, *Akebia trifoliata* NC029427 (Sun et al. 2016), *Decaisnea insignis* NC035941 (Li et al. 2017), *Passiflora caerulea* MT884000, and *Clematoclethra scandens* subsp. *actinidioides* OL457297.

## Data Availability

The genome sequence data that support the findings of this study are openly available in GenBank of NCBI at https://www.ncbi.nlm.nih.gov/ under the accession no. OL457297. The associated BioProject, SRA, and Bio-Sample numbers are PRJNA778432, SRR16841622, and SAMN22959467, respectively.
